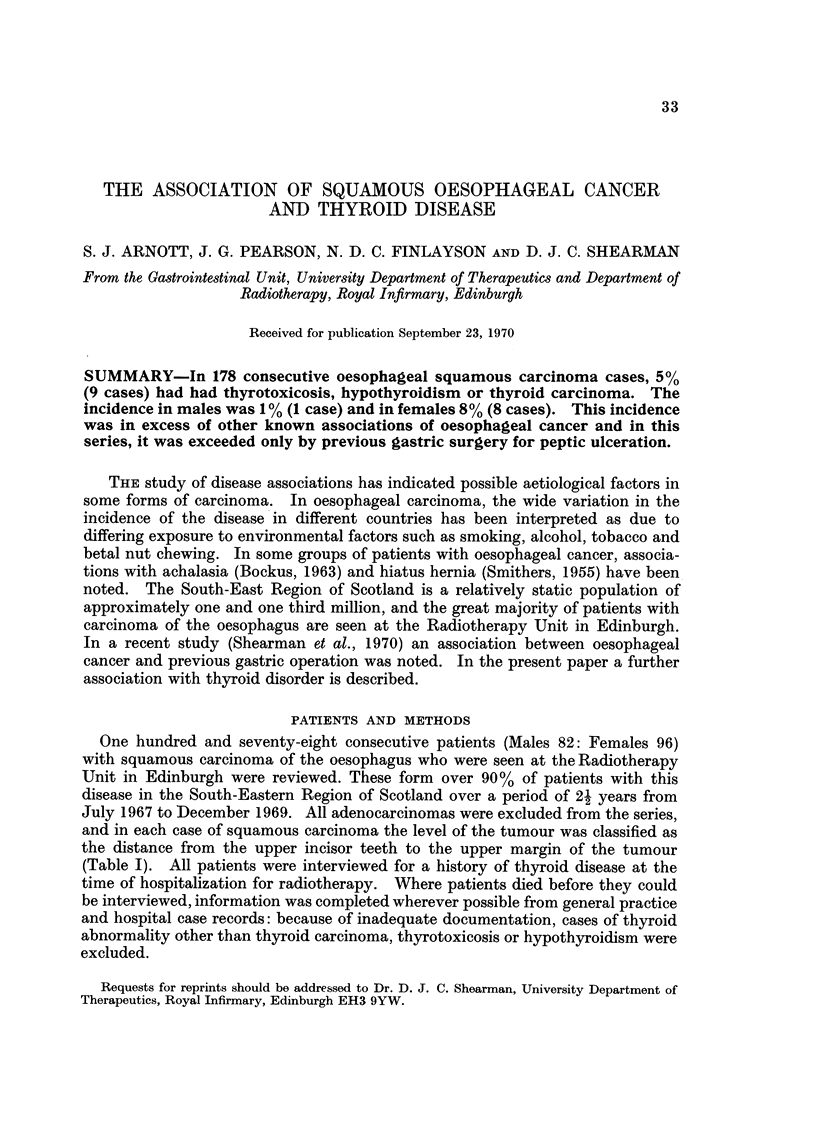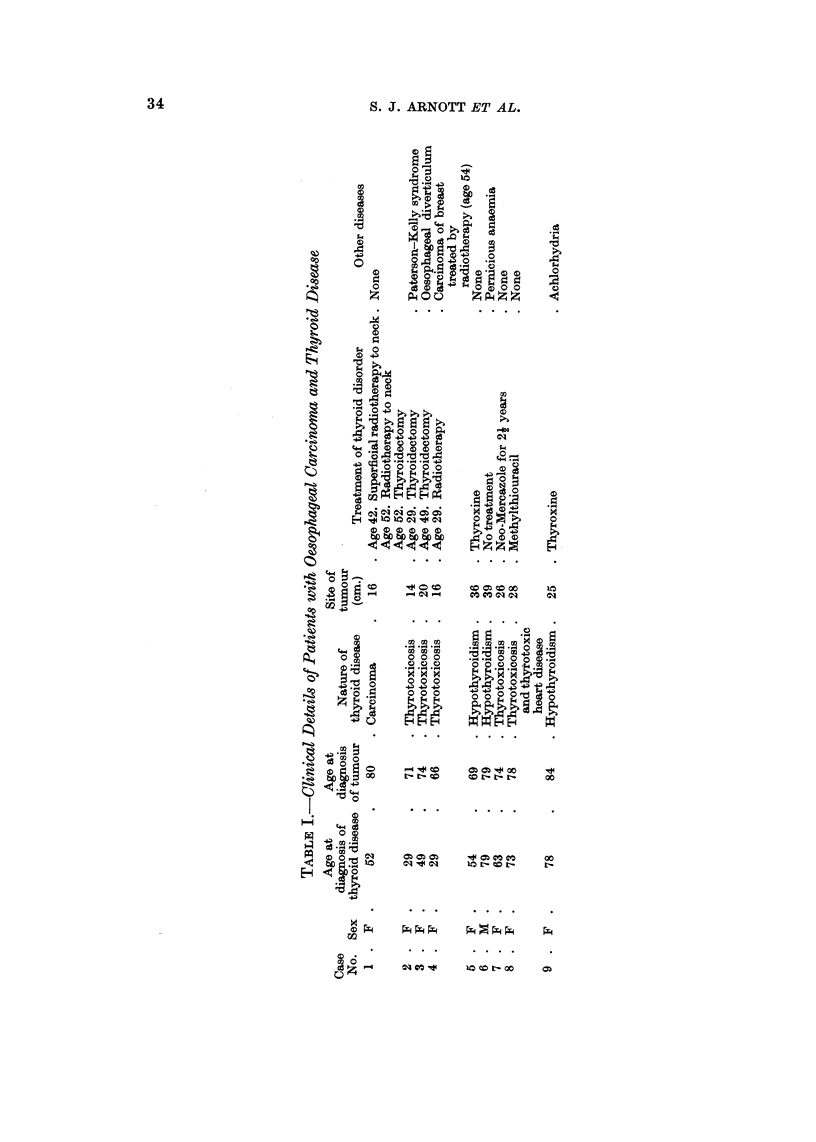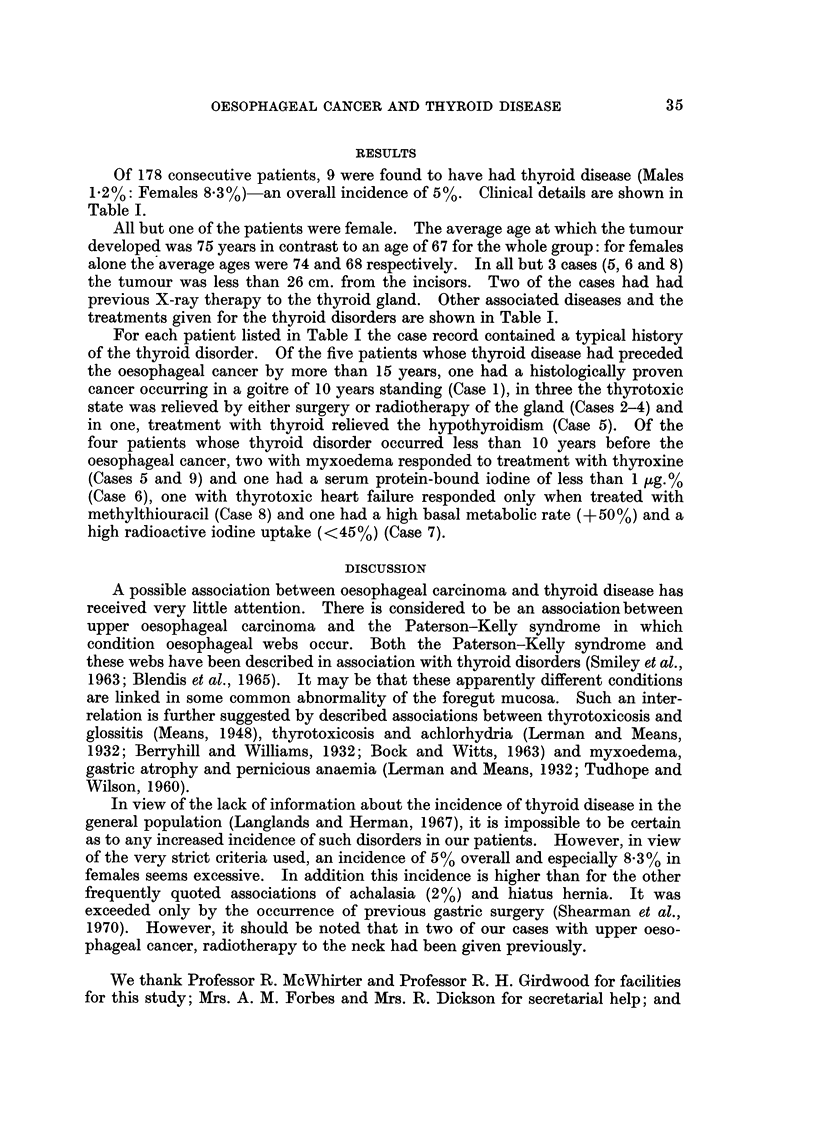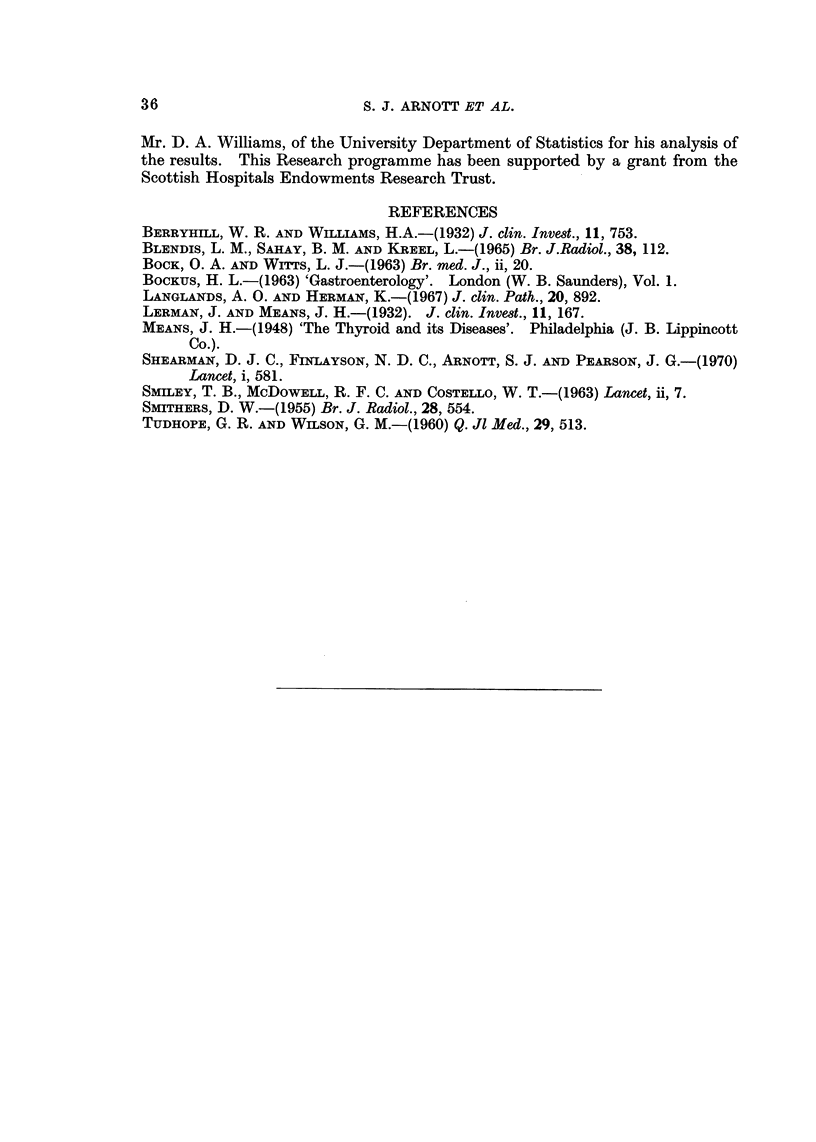# The Association of Squamous Oesophageal Cancer and Thyroid Disease

**DOI:** 10.1038/bjc.1971.5

**Published:** 1971-03

**Authors:** S. J. Arnott, J. G. Pearson, N. D. C. Finlayson, D. J. C. Shearman

## Abstract

In 178 consecutive oesophageal squamous carcinoma cases, 5% (9 cases) had had thyrotoxicosis, hypothyroidism or thyroid carcinoma. The incidence in males was 1% (1 case) and in females 8% (8 cases). This incidence was in excess of other known associations of oesophageal cancer and in this series, it was exceeded only by previous gastric surgery for peptic ulceration.


### THE ASSOCIATION OF SQUAMOUS OESOPHAGEAL CANCER AND THYROID DISEASE

S. J. ARNOTT, J. G. PEARSON, N. D. C. FINLAYSON AND D. J. C. SHEARMAN

*From the Gastrointestinal Unit, University Department of Therapeutics and Department of Radiotherapy, Royal Infirmary, Edinburgh*

Received for publication September 23, 1970

**SUMMARY—In 178 consecutive oesophageal squamous carcinoma cases, 5% (9 cases) had had thyrotoxicosis, hypothyroidism or thyroid carcinoma. The incidence in males was 1% (1 case) and in females 8% (8 cases). This incidence was in excess of other known associations of oesophageal cancer and in this series, it was exceeded only by previous gastric surgery for peptic ulceration.**

THE study of disease associations has indicated possible aetiological factors in some forms of carcinoma. In oesophageal carcinoma, the wide variation in the incidence of the disease in different countries has been interpreted as due to differing exposure to environmental factors such as smoking, alcohol, tobacco and betal nut chewing. In some groups of patients with oesophageal cancer, associations with achalasia (Bockus, 1963) and hiatus hernia (Smithers, 1955) have been noted. The South-East Region of Scotland is a relatively static population of approximately one and one third million, and the great majority of patients with carcinoma of the oesophagus are seen at the Radiotherapy Unit in Edinburgh. In a recent study (Shearman *et al.*, 1970) an association between oesophageal cancer and previous gastric operation was noted. In the present paper a further association with thyroid disorder is described.

### PATIENTS AND METHODS

One hundred and seventy-eight consecutive patients (Males 82: Females 96) with squamous carcinoma of the oesophagus who were seen at the Radiotherapy Unit in Edinburgh were reviewed. These form over 90% of patients with this disease in the South-Eastern Region of Scotland over a period of 2½ years from July 1967 to December 1969. All adenocarcinomas were excluded from the series, and in each case of squamous carcinoma the level of the tumour was classified as the distance from the upper incisor teeth to the upper margin of the tumour (Table I). All patients were interviewed for a history of thyroid disease at the time of hospitalization for radiotherapy. Where patients died before they could be interviewed, information was completed wherever possible from general practice and hospital case records: because of inadequate documentation, cases of thyroid abnormality other than thyroid carcinoma, thyrotoxicosis or hypothyroidism were excluded.

Requests for reprints should be addressed to Dr. D. J. C. Shearman, University Department of Therapeutics, Royal Infirmary, Edinburgh EH3 9YW.

TABLE I.—*Clinical Details of Patients with Oesophageal Carcinoma and Thyroid Disease*

| Case No. | Sex | Age at diagnosis of thyroid disease | Age at diagnosis of tumour | Nature of thyroid disease | Site of tumour (cm.) | Treatment of thyroid disorder | Other diseases |
|---|---|---|---|---|---|---|---|
| 1 | F | 52 | 80 | Carcinoma | 16 | Age 42. Superficial radiotherapy to neck<br>Age 52. Radiotherapy to neck<br>Age 52. Thyroidectomy | None |
| 2 | F | 29 | 71 | Thyrotoxicosis | 14 | Age 29. Thyroidectomy | Paterson–Kelly syndrome |
| 3 | F | 49 | 74 | Thyrotoxicosis | 20 | Age 49. Thyroidectomy | Oesophageal diverticulum |
| 4 | F | 29 | 66 | Thyrotoxicosis | 16 | Age 29. Radiotherapy | Carcinoma of breast treated by radiotherapy (age 54) |
| 5 | F | 54 | 69 | Hypothyroidism | 36 | Thyroxine | None |
| 6 | M | 79 | 79 | Hypothyroidism | 39 | No treatment | Pernicious anaemia |
| 7 | F | 63 | 74 | Thyrotoxicosis | 26 | Neo-Mercazole for $2\frac{1}{2}$ years | None |
| 8 | F | 73 | 78 | Thyrotoxicosis and thyrotoxic heart disease | 28 | Methylthiouracil | None |
| 9 | F | 78 | 84 | Hypothyroidism | 25 | Thyroxine | Achlorhydria |

### RESULTS

Of 178 consecutive patients, 9 were found to have had thyroid disease (Males 1·2%: Females 8·3%)—an overall incidence of 5%. Clinical details are shown in Table I.

All but one of the patients were female. The average age at which the tumour developed was 75 years in contrast to an age of 67 for the whole group: for females alone the average ages were 74 and 68 respectively. In all but 3 cases (5, 6 and 8) the tumour was less than 26 cm. from the incisors. Two of the cases had had previous X-ray therapy to the thyroid gland. Other associated diseases and the treatments given for the thyroid disorders are shown in Table I.

For each patient listed in Table I the case record contained a typical history of the thyroid disorder. Of the five patients whose thyroid disease had preceded the oesophageal cancer by more than 15 years, one had a histologically proven cancer occurring in a goitre of 10 years standing (Case 1), in three the thyrotoxic state was relieved by either surgery or radiotherapy of the gland (Cases 2–4) and in one, treatment with thyroid relieved the hypothyroidism (Case 5). Of the four patients whose thyroid disorder occurred less than 10 years before the oesophageal cancer, two with myxoedema responded to treatment with thyroxine (Cases 5 and 9) and one had a serum protein-bound iodine of less than 1 μg.% (Case 6), one with thyrotoxic heart failure responded only when treated with methylthiouracil (Case 8) and one had a high basal metabolic rate (+50%) and a high radioactive iodine uptake (<45%) (Case 7).

### DISCUSSION

A possible association between oesophageal carcinoma and thyroid disease has received very little attention. There is considered to be an association between upper oesophageal carcinoma and the Paterson–Kelly syndrome in which condition oesophageal webs occur. Both the Paterson–Kelly syndrome and these webs have been described in association with thyroid disorders (Smiley *et al.*, 1963; Blendis *et al.*, 1965). It may be that these apparently different conditions are linked in some common abnormality of the foregut mucosa. Such an inter-relation is further suggested by described associations between thyrotoxicosis and glossitis (Means, 1948), thyrotoxicosis and achlorhydria (Lerman and Means, 1932; Berryhill and Williams, 1932; Bock and Witts, 1963) and myxoedema, gastric atrophy and pernicious anaemia (Lerman and Means, 1932; Tudhope and Wilson, 1960).

In view of the lack of information about the incidence of thyroid disease in the general population (Langlands and Herman, 1967), it is impossible to be certain as to any increased incidence of such disorders in our patients. However, in view of the very strict criteria used, an incidence of 5% overall and especially 8·3% in females seems excessive. In addition this incidence is higher than for the other frequently quoted associations of achalasia (2%) and hiatus hernia. It was exceeded only by the occurrence of previous gastric surgery (Shearman *et al.*, 1970). However, it should be noted that in two of our cases with upper oesophageal cancer, radiotherapy to the neck had been given previously.

We thank Professor R. McWhirter and Professor R. H. Girdwood for facilities for this study; Mrs. A. M. Forbes and Mrs. R. Dickson for secretarial help; and

Mr. D. A. Williams, of the University Department of Statistics for his analysis of the results. This Research programme has been supported by a grant from the Scottish Hospitals Endowments Research Trust.

### REFERENCES

BERRYHILL, W. R. AND WILLIAMS, H.A.—(1932) *J. clin. Invest.*, **11**, 753.
BLENDIS, L. M., SAHAY, B. M. AND KREEL, L.—(1965) *Br. J.Radiol.*, **38**, 112.
BOCK, O. A. AND WITTS, L. J.—(1963) *Br. med. J.*, ii, 20.
BOCKUS, H. L.—(1963) 'Gastroenterology'. London (W. B. Saunders), Vol. 1.
LANGLANDS, A. O. AND HERMAN, K.—(1967) *J. clin. Path.*, **20**, 892.
LERMAN, J. AND MEANS, J. H.—(1932). *J. clin. Invest.*, **11**, 167.
MEANS, J. H.—(1948) 'The Thyroid and its Diseases'. Philadelphia (J. B. Lippincott Co.).
SHEARMAN, D. J. C., FINLAYSON, N. D. C., ARNOTT, S. J. AND PEARSON, J. G.—(1970) *Lancet*, i, 581.
SMILEY, T. B., MCDOWELL, R. F. C. AND COSTELLO, W. T.—(1963) *Lancet*, ii, 7.
SMITHERS, D. W.—(1955) *Br. J. Radiol.*, **28**, 554.
TUDHOPE, G. R. AND WILSON, G. M.—(1960) *Q. Jl Med.*, **29**, 513.